# Plasma nitrite concentrations decrease after hyperoxia-induced oxidative stress in healthy humans

**DOI:** 10.1186/2050-6511-13-S1-A88

**Published:** 2012-09-17

**Authors:** Darko Modun, Mladen Krnić, Jonatan Vuković, Višnja Kokić, Lea Kukoč-Modun, Dimitrios Tsikas, Zeljko Dujić

**Affiliations:** 1 Department of Pharmacology, School of Medicine, University of Split, 21000 Split, Croatia; 2 Department of Endocrinology, University Hospital Split, 21000 Split, Croatia; 3 Department of Analytical Chemistry, Faculty of Chemistry and Technology, University of Split, 21000 Split, Croatia; 4 Institute of Clinical Pharmacology, Hannover Medical School, 30625 Hannover, Germany; 5 Department of Physiology, School of Medicine, University of Split, 21000 Split, Croatia

## Background

We measured plasma nitrite, the biochemical marker of endothelial nitric oxide (^•^NO) synthesis, before and after hyperoxia, in order to test the hypothesis that hyperoxia-induced vasoconstriction is a consequence of reduced bioavailability of ^•^NO due to elevated oxidative stress.

## Methods

Ten healthy males breathed 100% normobaric O_2_ for 30 min between the 15^th^ and 45^th^ min of the 1 h study protocol. Plasma nitrite and malondialdehyde (MDA), arterial stiffness (indicated by augmentation index, AIx) and arterial oxygen (P_tc_O_2_) pressure were measured in the 1^st^, 15^th^, 45^th^ and 60^th^ minute of the study.

## Results

Breathing of normobaric 100% oxygen during 30 min caused an increase of P_tc_O_2_ (from 75 ± 2 to 412 ± 25 mm Hg), AIx (from −63 ± 4 to −51 ± 3%) and MDA (from 152 ± 13 to 218 ± 15 nmol/L) and a decrease in plasma nitrite (from 918 ± 58 to 773 ± 55 nmol/L). During the 15-min recovery phase the plasma nitrite, AIx and MDA values remained altered.

## Conclusions

This study suggests that the underlying mechanism of hyperoxia-induced vasoconstriction may result from reduced ^•^NO bioavailability due to elevated and sustained oxidative stress.

